# Mortality in pediatric oncology and stem cell transplant patients with bloodstream infections

**DOI:** 10.3389/fonc.2022.1063253

**Published:** 2023-01-11

**Authors:** Daniel N. Willis, Mary Claire McGlynn, Patrick J. Reich, Robert J. Hayashi

**Affiliations:** ^1^ Department of Pediatrics, Division of Hematology/Oncology, Washington University School of Medicine, St. Louis Children’s Hospital, St. Louis, MO, United States; ^2^ Department of Pediatrics, Washington University School of Medicine, St. Louis Children’s Hospital, St. Louis, MO, United States; ^3^ Department of Pediatrics, Division of Infectious Diseases, Washington University School of Medicine, St. Louis Children’s Hospital, St. Louis, MO, United States

**Keywords:** pediatrics, oncology, bloodstream infection, mortality, stem cell transplantation

## Abstract

**Background:**

Bloodstream infections (BSI) continue to represent a significant source of morbidity for pediatric oncology patients, however less is known regarding this population’s risk of death. We sought to evaluate the risk of BSI and death at a large pediatric cancer center.

**Methods:**

We retrospectively collected inpatient data from pediatric oncology and hematopoietic stem cell transplant (HSCT) patients over a 9-year period. We performed univariate and multivariable modeling to assess risk of BSI and mortality examining the following variables: demographics, underlying malignancy, history of HSCT, central line type, and febrile neutropenia (FN).

**Results:**

During the study period, 6763 admissions from 952 patients met inclusion criteria. BSI occurred in 367 admissions (5.4%) from 231 unique individuals. Risk factors for BSI include younger age, diagnoses of hemophagocytic lymphohistiocytosis or acute myeloid leukemia, ethnicity, and history of HSCT. Mortality for those with BSI was 6.5%, compared to 0.7% without (OR 7.2, CI 4.1 – 12.7, p<0.0001). In patients with BSI, admissions with FN were associated with reduced mortality compared to admissions without FN (OR 0.21, CI 0.05 – 0.94, p=0.04). In both univariate and multivariable analysis, no other risk factor was significantly associated with mortality in patients with BSI.

**Conclusion:**

BSI is a significant source of mortality in pediatric oncology and HSCT patients. While demographic variables contribute to the risk of BSI, they did not influence mortality. These findings highlight the importance of BSI prevention to reduce the risk of death in pediatric oncology patients. Future studies should focus on comprehensive BSI prevention.

## 1 Introduction

Bloodstream infections (BSI) continue to represent a significant source of morbidity for pediatric oncology patients (1). Modern cancer treatment regimens are often intensive with associated morbidity and mortality. Amongst children who die of cancer within 5 years of diagnosis, 75% will die of disease progression and 21.4% will die of treatment related mortality (1, 2). Infections of any kind account for 16% of all deaths (3).

BSI has long been known to be a risk factor for death in oncology patients. In adults, case-fatality rates have been reported as high as 32% (4). Although pediatric patients are at lower risk for BSI than adults, it remains a significant problem (5). Multiple factors contribute to the risk for BSI. Intensive chemotherapy and radiation impairs the immune system. Patients undergoing hematopoietic stem cell transplantation (HSCT) with its accompanying immunosuppression are also at substantial risk, and those with underlying hematologic malignancies are at highest risk (6). Patients with neutropenia are especially at increased risk of BSI (7), with sepsis rates as high as 16% (8). Finally, the vast majority of pediatric oncology patients possess central venous catheters (CVCs) which disrupt the body’s physical barrier against BSI. Both external lines and non-tunneled lines have been shown to increase the risk of BSI (9, 10). Some reports have examined the risk of mortality in selected patient populations, primarily HSCT. Studies in HSCT have shown increased risk of infection related mortality in adults compared to children and following allogenic transplant (11). Enterococcus infections have been demonstrated to confer increased risk of mortality in both pediatric and adult HSCT patients (12). Little is known with regard to the risk of death from BSI across the general pediatric cancer population, although one study described risk in pediatric acute lymphoblastic leukemia, showing trisomy 21 to be a risk factor (13). This report examines the risk of death for hospitalized pediatric cancer patients with BSI and associated risk factors. We hypothesized modifiable risk factors will emerge that can be mediated to reduce mortality.

## 2 Materials and methods

### 2.1 Patient population

In this retrospective cohort study, we evaluated pediatric oncology patients who were admitted to a 400-bed free-standing children’s hospital from July 2010 through February 2018. This study was approved by the institutional review board (IRB 201710165). All patients with a diagnosed malignancy admitted to the pediatric oncology service were included. Diagnoses were determined by ICD9/10 code and validated by chart review. Data was analyzed with each admission representing a separate data point. HSCT admissions were defined as any admission in which a transplant procedure occurred or any subsequent admission for that patient. In the event that a patient received multiple transplant procedures, the admissions were coded according to the first transplant received. Fever and neutropenia diagnoses were determined by any billing code for ICD9/10 code (780.61/R50.81) during the admission. Death was determined by patient being designated as “deceased” in the medical record. There were separate inpatient and outpatient medical records at the time of this study and both were queried. If data was missing, a manual review was performed to verify vital status.

Occurring at a single institution, our prophylactic and treatment antibiotic practices were relatively uniform and institutional standards did not change during the study period. For neutropenic patients, an antipseudomonal cephalosporin was considered first line for empiric treatment of sepsis. Vancomycin was added if the patient was critically ill or had persistent fever beyond 48 hours. Antibiotics were not discontinued until afebrile and the patient demonstrated evidence of bone marrow recovery.

### 2.2 Outcome measure

BSIs were identified using any positive blood cultures documented in the medical record during hospitalization. The date a positive blood culture was obtained and organism isolated from it was documented. In the event that organisms grew on follow up blood cultures, only the initial positive culture was utilized for analysis. Additionally, if multiple unique positive cultures occurred during an admission, the first such episode was used for evaluation.

### 2.3 Statistical analysis

All data analyses were conducted by SAS^®^ (SAS Institute Inc., Cary, NC, USA) 9.4 version. Univariate mixed effects logistic regression model was performed to assess the effect of risk factor on outcome. Odds ratio and 95% confidence interval were presented to measure the magnitude of the effect. A P-value < 0.05 was considered a significant result. This analysis was also performed on the subgroup of patients who died during admission, to assess for the effect of risk factors on mortality. All missing data or unknown data was excluded from the analysis.

## 3 Results

During the study period, 6763 admissions met criteria for inclusion from 952 unique patients. **Table 1** displays the characteristics of these admissions. Age at time of diagnosis ranged from under a year to up to 24 years old, with a median age of 8.8 years. The ethnicity classification reflects the underlying population of this referral region. Solid tumor patients accounted for 45% of all admissions. Of all admissions, 23% were constituted by HSCT patients. Febrile neutropenia (FN) was noted in 20.8% of all admissions. Only 1% of admissions resulted in death.

**Table 1:** Characteristics of pediatric oncology patients and admissions during the study period.

	Patients	Admissions	Admissions with BSI
Total	952	6763	367
Median Age in years (interquartile range)	n/a	8.8 (3.9, 14.4)	6.1 (2.8, 13.1)
Diagnosis of Disease			
*CNS*	177 (18.6%)	1011 (15%)	42 (11.4%)
*Histiocytosis*	16 (1.7%)	48 (0.7%)	7 (1.9%)
*Leukemia*	284 (29.8%)	1963 (29%)	164 (44.7%)
*Lymphoma*	132 (13.9%)	662 (9.8%)	23 (6.3%)
*Solid Tumor*	337 (35.4%)	3039 (44.9%)	128 (34.9%)
*Other*	6 (0.6%)	40 (0.6%)	3 (0.8%)
Race			
*African American*	132 (13.9%)	927 (13.7%)	46 (12.5%)
*Caucasian*	762 (80.0%)	5473 (80.9%)	298 (81.2%)
*Asian*	9 (1.0%)	59 (0.9%)	2 (0.5%)
*Unknown*	49 (5.1%)	304 (4.5%)	21 (5.7%)
Ethnicity			
*Hispanic or Latino*	9 (0.9%)	65 (1%)	3 (0.8%)
*Not Hispanic or Latino*	741 (77.8%)	5176 (76.5%)	303 (82.6%)
*Unreported*	202 (21.2%)	1522 (22.5%)	61 (16.6%)
HSCT	159 (16.7%)	1569 (23.2%)	174 (47.4%)
*Allogenic*	78 (49.1%)	706 (45%)	109 (62.6%)
*Autologous*	81 (50.9%)	863 (55%)	65 (37.4%)
Died during admission			
*Yes*	n/a	67 (1%)	24 (6.5%)
*No*	n/a	6696 (99%)	343 (93.5%)
FN Diagnosis Code			
*Yes*	n/a	1404 (20.8%)	139 (37.9%)
*No*	n/a	5359 (79.2%)	228 (62.1%)

CNS, central nervous system; HSCT, hematopoietic stem cell transplantation; FN, Febrile Neutropenia.

BSI occurred in 367 admissions representing an incidence of 5.4% from 231 unique patients. Organisms identified were diverse with 60.5% of infections caused by Gram-positive organisms, 33% by Gram-negative organisms, 2.5% by anaerobes and 3.5% by fungi (**Table 2**). The most common species isolated were *S. epidermidis* (19.1%) and other coagulase-negative staphylococci (10.6%). Sensitivities were performed on 39 of 70 isolates of *S. epidermidis* with methicillin-resistant *S. epidermidis* (MRSE) present in 31 of 39 (79.4%) isolates. For *S. aureus*, 8 of 24 (33.3%) were methicillin-resistant *S. aureus* (MRSA). All *S. aureus*, *coagulase-negative staphylococci*, and viridans group Strep isolates were sensitive to vancomycin. Of the 13 Enterococci isolates, 3 (23%) were vancomycin resistant. Of gram negative bacilli, only 9 of 143 (6.3%) were resistant to cefepime. Characterization of the source of the positive blood culture revealed that 50.7% were collected from patients with external tunneled lines, 18.8% from port-a-caths, and 6% from peripherally inserted central (PICC) lines. Peripheral cultures were the source of 7.4% of BSI and 17.2% were collected from unspecified sites.

**Table 2:** Characteristics of positive blood cultures (n=367).

Blood Culture Source	n (%)
External Central Line	186 (50.7)
Port-a-cath	69 (18.8)
PICC	22 (6)
Peripheral	27 (7.4)
Unspecified	63 (17.1)
Organism Isolated	n (%)
**Gram-positive organisms**	**222 (60.5)**
*Staphylococcus epidermidis*	70 (19.1%)
Other coagulase negative *Staphylococcus* species	39 (10.6%)
Viridans Group Streptococci	29 (7.9%)
*Staphylococcus aureus*	24 (6.5)
*Bacillus* species	19 (5.2%)
*Enterococcus faecalis*	6 (1.6%)
*Streptococcus pneumoniae*	5 (1.4%)
Other Gram-positive species	30 (8.2%)
**Gram-negative**	**121 (33%)**
*Escherichia coli*	27 (7.4%)
*Klebsiella pneumoniae*	19 (5.2%)
*Enterobacter cloacae* complex	12 (3.3%)
*Klebsiella oxytoca*	7 (1.9%)
*Pseudomonas aeruginosa*	8 (2.2%)
Other *Pseudomonas* species	6 (1.6%)
*Acinetobacter calcoaceticus-baumanii* complex	6 (1.6%)
Other Gram-negative species	36 (9.8%)
**Anaerobes**	**9 (2.5%)**
*Clostridium* species	5 (1.4%)
Other anaerobes	4 (1.1%)
**Acid fast**	**2 (0.5%)**
Non-tuberculosis mycobacteria	2 (0.5%)
**Fungus**	**13 (3.5%)**
*Candida albicans*	3 (0.8%)
Other *Candida* species	8 (2.2%)
Other fungi	2 (0.5%)

PICC, peripherally inserted central catheter. Bold values equals statistically significant (p<0.05).

### 3.1 Risk factors for BSI

Several factors were significantly associated with BSI on univariate (**Table 3**) and multivariable analysis (**Table 4**). Admissions with BSI were more likely to occur in younger patients (OR 0.96, 95%CI 0.94-0.98, p<0.0001) and less likely in patients in whom ethnicity was not reported (0.7 95%CI 0.5-0.97, p=0.0275). BSI was associated with diagnoses of acute myeloid leukemia (AML) (OR 3.0, 95%CI 1.9-5.0, p<0.0001), and hemophagocytic lymphohistiocytosis (HLH) (OR 3.3, 95%CI 1.2-9.3, p=0.0232). The diagnosis of FN was present in 38% of admissions with BSI. Admission with FN was associated with an increased risk for BSI with an odds ratio 2.2 times higher compared to non-BSI admissions (95%CI 1.7-2.7, p<0.0001). In subset analysis of admissions with FN, age and mortality was no longer statistically associated with BSI. Patients who had undergone HSCT of any type were also at increased risk for BSI. Within the HSCT cohort, those admissions following allogeneic transplant were slightly more likely associated with BSI compared to autologous HSCT admissions (allogenic OR 2.9, 95%CI 2.1-4.1 with p<0.0001 verses autologous OR 2.3, 95%CI 1.7-3.2 with p<0.0001).

**Table 3:** Univariate analysis of risk factors associated with positive blood cultures during admission (n=6763).

	Positive (N=367)	Negative (N=6396)	P-Value	Odds Ratio (95% CI)
**Median Age at Admission (years)**	6.1 (2.8 – 13.1)	8.8 (4.0 – 14.5)	**0.0003**	**0.97 (0.95 – 0.98)**
Diagnosis of Disease				
*CNS*	42 (11.4%)	969 (15.2%)		Reference
**HLH**	**6 (1.6%)**	**18 (0.3%)**	**<0.0001**	**7.7 (2.9 - 20.4)**
LCH	1 (0.3%)	23 (0.4%)	0.9976	1.0 (0.1 - 7.6)
ALL	92 (25.1%)	1516 (23.7%)	0.0781	1.4 (0.9 - 2.0)
**AML**	**63 (17.2%)**	**218 (3.4%)**	**<0.0001**	**6.7 (4.4 - 10.1)**
**Other Leukemia**	**9 (2.5%)**	**65 (1%)**	**0.0029**	**3.2 (1.5 - 6.9)**
Lymphoma	23 (6.3%)	639 (10%)	0.4826	0.8 (0.5 - 1.4)
Solid Tumor	128 (34.9%)	2911 (45.5%)	0.9370	1.0 (0.7 - 1.5)
Other	3 (0.8%)	37 (0.6%)	0.3135	1.9 (0.6 - 6.3)
Race				
*African American*	46 (12.5%)	881 (13.8%)	0.5472	0.9 (0.7 – 1.2)
*Caucasian*	298 (81.2%)	5175 (80.9%)		Reference
*Asian*	2 (0.5%)	57 (0.9%)	0.4928	0.6 (0.1 – 2.5)
*Unknown*	21 (5.7%)	283 (4.4%)	0.2787	1.3 (0.8 – 2.0)
Ethnicity				
*Hispanic or Latino*	3 (0.8%)	62 (1%)	0.6731	0.8 (0.2 – 2.5)
*Not Hispanic or Latino*	303 (82.6%)	4873 (76.2%)		Reference
*Unreported*	61 (16.6%)	1461 (22.8%)	**0.0056**	**0.7 (0.5 – 0.9)**
HSCT during admission or post-HSCT admission				
*Yes*	174 (47.4%)	1395 (21.8%)	**<0.0001**	**3.2 (2.6 – 4.0)**
*No*	193 (52.6%)	5001(78.2%)		Reference
HSCT Transplant Type				
*Allogenic*	109 (62.6%)	597 (42.8%)	**<0.0001**	**2.2 (1.6 – 3.1)**
*Autologous*	65 (37.4%)	798 (57.2%)		Reference
Died during admission				
*Yes*	24 (6.5%)	43 (0.7%)	**<0.0001**	**10.3 (6.1 – 17.4)**
*No*	343 (93.5%)	6353 (99.3%)		Reference
Fever and Neutropenia Diagnosis Code				
*Yes*	139 (37.9%)	1265 (19.8%)	**<0.0001**	**2.5 (2.0 – 3.1)**
*No*	228 (62.1%)	5131 (80.2%)		Reference

CNS, central nervous system; HLH, hemophagocytic lymphohistiocytosis; LCH, Langerhans cell histiocytosis; ALL, acute lymphoblastic leukemia; AML, acute myeloid leukemia; HSCT, hematopoietic stem cell transplantation. Bold values equals statistically significant (p<0.05).

**Table 4:** Multivariable analysis of risk factors associated with positive blood cultures during admission (n=6763).

	Odds Ratio (95% CI)	P-Value
**Age at admission (year), per 1-year increase**	**0.96 (0.94 – 0.98)**	**<0.0001**
Diagnosis of disease		
*CNS*	Reference	
***HLH***	**3.3 (1.2 – 9.3)**	**0.0232**
*LCH*	0.5 (0.1 – 4.0)	0.5384
*ALL*	1.4 (0.7 – 1.5)	0.8567
***AML***	**3.0 (1.9 – 5.0)**	**<0.0001**
*Other Leukemia*	1.4 (0.6 – 3.2)	0.4209
*Lymphoma*	0.9 (0.5 – 1.5)	0.7238
*Solid Tumor*	1.0 (0.7 – 1.4)	0.8194
*Other*	1.1 (0.3 – 3.9)	0.8777
Race		
*African American*	1.1 (0.8 – 1.5)	0.7202
*Caucasian*	Reference	
*Asian*	0.5 (0.1 – 2.1)	0.3516
*Unknown*	1.4 (0.9 – 2.3)	0.1823
Ethnicity		
*Hispanic or Latino*	0.8 (0.2 – 2.6)	0.6493
*Not Hispanic or Latino*	Reference	
***Unreported***	**0.7 (0.5 – 0.97)**	**0.0275**
HSCT during admission or post HSCT admission		
***Allogenic***	**2.9 (2.1 – 4.1)**	**<0.0001**
***Autologous***	**2.3 (1.7 – 3.2)**	**<0.0001**
*No Transplant*	Reference	
Died during admission		
***Yes***	**7.2 (4.1 – 12.7)**	<0.0001
*No*	Reference	
Fever and Neutropenia Diagnosis Code		
***Yes***	**2.2 (1.7 – 2.7)**	**<0.0001**
*No*	Reference	

CNS, central nervous system; HLH, hemophagocytic lymphohistiocytosis; LCH, Langerhans cell histiocytosis; ALL, acute lymphoblastic leukemia; AML, acute myeloid leukemia; HSCT, hematopoietic stem cell transplantation. Bold values equals statistically significant (p<0.05).

### 3.2 Mortality

During this study period, there were a total of 67 inpatient deaths, representing 7% of patients. Death was strongly associated with BSI, with 6.5% of admissions with BSI resulting in death compared to 0.7% of admissions without BSI (OR 7.2, CI 4.1 – 12.7, p<0.0001) (**Table 4**). On multivariable analysis within admissions with BSI, risk of death was not significantly associated with age, diagnosis, history of HSCT, race or ethnicity. The risk of death was not influenced by intravenous line type (**Table 5**). Surprisingly, admissions with FN were associated with a lower risk of death (OR 0.21 CI 0.05 – 0.94, p=0.04) (**Table 6**).

**Table 5:** Univariate Analysis of Risk Factors Associated with Mortality during Admissions with BSI (N=367).

		Died (N=24)	Alive (N=343)	P-Value	Odds Ratio (95% CI)
Median Age at Admission (years)		10.9 (4.2 – 16.9)	6.0 (2.8 – 12.9)	0.0536	1.07 (1.0 – 1.14)
Diagnosis of Disease					
*CNS*		2 (8%)	40 (11.7%)		Reference
*HLH*		0 (0%)	6 (1.7%)	>0.99	N/A
*LCH*		0 (0%)	1 (0.3%)	>0.99	N/A
*ALL*		5 (21%)	87 (25.4%)	0.8716	1.15 (0.21 – 6.27)
*AML*		10 (42%)	53 (15.5%)	0.1003	3.77 (0.77 – 18.43)
*Other Leukemia*		1 (4%)	8 (2.3%)	0.4776	2.50 (0.20 – 31.65)
*Lymphoma*		2 (8%)	21 (6.1%)	0.5356	1.91 (0.25 – 14.75)
*Solid Tumor*		3 (13%)	125 (36.4%)	0.4325	0.48 (0.08 – 3.02)
*Other*		1 (4%)	2 (0.6%)	0.1080	10 (0.60 – >100)
Race					
*African American*		1 (4%)	45 (13.1%)	0.2846	0.33 (0.04 – 2.55)
*Caucasian*		19 (79%)	279 (81.3%)		Reference
*Asian*		1 (4%)	1 (0.3%)	0.0637	14.68 (0.86 – >100)
*Unknown*		3 (13%)	18 (5.3%)	0.1835	2.45 (0.65 – 9.18)
Ethnicity					
*Hispanic or Latino*		0 (0%)	3 (0.9%)	>0.99	N/A
*Not Hispanic or Latino*		22 (92%)	281 (81.9%)		Reference
*Unreported*		2 (8%)	59 (17.2%)	0.2670	0.43 (0.10 – 1.91)
HSCT during admission or post-HSCT admission					
*Yes*	11 (46%)		163 (47.5%)	0.8733	0.93 (0.41 – 2.16)
*No*	13 (54%)		180 (52.5%)		Reference
HSCT Transplant Type					
*Allogenic*	10 (91%)		99 (60.7%)	0.0841	6.47 (0.77 – 54.06)
*Autologous*	1 (9%)		64 (39.3%)		Reference
Blood Culture Source					
*External Central Line*	16 (67%)		170 (49.6%)		Reference
*Port-a-cath*	4 (17%)		65 (19%)	0.4518	0.65 (0.21 – 2.02)
*PICC*	3 (12%)		19 (5.5%)	0.4324	1.68 (0.45 – 6.25)
*Peripheral*	0 (0%)		27 (7.9%)	>0.99	N/A
*Unspecified*	1 (4%)		62 (18.1%)	0.0872	0.17 (0.02 – 1.31)
Fever and Neutropenia Diagnosis Code					
*Yes*	4 (17%)		135 (39.4%)	**0.0422**	**0.31 (0.10 – 0.96)**
*No*	20 (83%)		208 (60.6%)		Reference

CNS, central nervous system; HLH, hemophagocytic lymphohistiocytosis; LCH, Langerhans cell histiocytosis; ALL, acute lymphoblastic leukemia; AML, acute myeloid leukemia; HSCT, hematopoietic stem cell transplantation; PICC, peripherally inserted central catheter. Bold values equals statistically significant (p<0.05).

**Table 6:** Multivariable Analysis of Risk Factors Associated with Mortality during Admissions with BSI (N=367).

	Odds Ratio (95% CI)	P-Value
Age at admission (year), per 1-year increase	1.02 (0.92 – 1.14)	0.7259
Diagnosis of disease		
*CNS*	Reference	
*HLH*	N/A	>0.99
*LCH*	N/A	>0.99
*ALL*	0.90 (0.07 – 10.85)	0.9325
*AML*	4.58 (0.39 – 53.69)	0.2248
*Other Leukemia*	4.73 (0.13 – >100)	0.3972
*Lymphoma*	2.15 (0.11 – 41.61)	0.6109
*Solid Tumor*	0.49 (0.04 – 5.44)	0.5595
*Other*	11.03 (0.14 – >100)	0.2835
Race		
*African American*	0.20 (0.01 – 2.84)	0.2336
*Caucasian*	Reference	
*Asian*	29.07 (0.37 – >100)	0.1301
*Unknown*	4.67 (0.59 – 36.88)	0.1433
Ethnicity		
*Hispanic or Latino*	N/A	>0.99
*Not Hispanic or Latino*	Reference	
*Unreported*	0.25 (0.03 – 1.95)	0.1848
HSCT Transplant type during admission or post-HSCT admission^1^		
*Allogenic*	0.51 (0.11 – 2.39)	0.3870
*Autologous*	0.23 (0.02 – 3.42)	0.2833
*No Transplant*	Reference	
Blood Culture Source		
*External Central Line*	Reference	
*Port-a-cath*	0.58 (0.10 – 3.54)	0.5493
*PICC*	0.66 (0.09 – 4.94)	0.6804
*Peripheral*	N/A	>0.99
*Unspecified*	0.15 (0.01 – 2.84)	0.2024
Fever and Neutropenia Diagnosis Code		
*Yes*	**0.21 (0.05 – 0.94)**	**0.0412**
*No*	Reference	

^1^Combined transplant (yes/no) and transplant type to make the covariate covering all samples and be mutually exclusive.

CNS, central nervous system; HLH, hemophagocytic lymphohistiocytosis; LCH, Langerhans cell histiocytosis; ALL, acute lymphoblastic leukemia; AML, acute myeloid leukemia; HSCT, hematopoietic stem cell transplantation; PICC, peripherally inserted central catheter. Bold values equals statistically significant (p<0.05).

## 4 Discussion

This study provides one of the largest cohort studies evaluating mortality in hospitalized pediatric oncology patients associated with BSI. Perhaps not surprisingly, studies in adult HSCT patients have demonstrated risk of mortality associated with presence of shock and inappropriate empiric antibiotic regimen. But while BSI remains a significant problem in pediatric oncology patients, the risk of mortality has not been extensively examined and this study is the first to our knowledge that has evaluated risk for mortality in pediatric patients with BSI in the pediatric oncology population. We found that while patients with FN were more likely to be associated with BSI, the majority of admissions with BSI were not associated with admissions with FN. This emphasizes the importance of mitigation strategies for addressing BSI for the entire pediatric oncology population and not just FN.

Importantly, we found that BSI is strongly associated with mortality. In patients who develop BSI, the risk of death is not influenced by underlying malignancy, race, ethnicity, or history of transplant. Surprisingly, we found that admissions with FN are associated with a reduced risk of death, despite its association with an elevated risk for BSI. While speculative, this unexpected result may reflect the heightened vigilance and prompt care typically delivered to patients with FN with their known susceptibility for sepsis. Previous reports have supported that prompt antibiotic administration decreases risk of death (14). Our institutional practice of the prompt administration (within 60 minutes of fever onset) of empiric antipseudomonal cephalosporins or equivalent spectrum antibiotics in patients with FN and its continuation until neutrophil count recovery may influence mortality from BSI in this population. To accomplish this, the initial dose is often given after obtaining cultures, but prior to lab results, with a presumption of neutropenia until proven otherwise. Despite the concern for prolonged empiric antibiotic administration, we saw little antibacterial resistance, with no vancomycin resistant *S. aureus* or *viridans group Strep* isolates and only 6% cefepime resistance in gram negative bacilli. In patients with BSI who subsequently died, only 2 of 24 grew organisms resistant to cefepime. Thus our current practice does not appear to jeopardize containing antibiotic resistant organisms in our patient population.

Our prophylactic and empiric antibiotic practices did not change during the study period. All patients received pneumocystis prophylaxis. Anti-candidal and anti-bacterial prophylaxis were provided to HSCT patients. In this environment with relatively consistent and uniform antibiotic practice, this study indicates that BSI conveys a high risk of mortality independent of demographics or underlying malignancy and, currently, there is no identifiable risk factor that can be targeted, other than the presence of BSI to mitigate this risk. Multiple interventions have been implemented to reduce the risk of BSI by many groups. Central line maintenance recommendations have been effective to some degree in reducing BSI in pediatric centers (15). Antibiotic prophylaxis has also been used in high-risk oncology populations with measurable effect, although that effect is not seen in all populations, including in HSCT, where BSI risk remains substantial (16, 17). Despite such efforts, there continues to be a significant risk for BSI associated mortality. Comprehensive efforts for BSI prevention are critical to reduce the risk of mortality in this population. Success in reducing the incidence of BSI could lead to substantive improvements in the overall survival of pediatric oncology patients.

The bacterial species distribution observed is in accordance with prior reports of common organisms in pediatric cancer patients (18). Although numbers are small, death did not occur predominantly in any particular bacterial grouping, indicating that any BSI is a risk for death.

We identified multiple variables that increased the risk for BSI. We found younger age to be associated with an increased risk for infection when analyzed as a continuous variable. Patients with HLH, AML, and HSCT were all at increased risk for BSI. The degree of immunosuppression for a patient has been long associated with an increased risk of infection and patients with HLH, AML and HSCT all represent patients receiving highly immunosuppressive therapy (7). Non-reported ethnicity was also protective against BSI. Ethnicity data was based on self-reported demographic information. While some demographic groups are less likely to report ethnicity, or identify in a single category, additional studies are needed to provide further clarity on the role of ethnicity on the risk of BSI (19).

This study has several limitations. The study is retrospective which limits our ability to draw conclusions regarding these associations. The population is also heterogeneous and while we stratified to evaluate for this, we recognize that different disease groups are associated with different risk for mortality. The study includes episodes of BSI that occurred both upon admission or which developed several days after admission to the hospital. These two groups may represent clinically different populations with different co-morbidities. The cohort also included all pediatric oncology admissions regardless of reason for admission. This included scheduled admissions for blood products and routine chemotherapy, and admissions for therapy associated toxicity and illness. This study also examined inpatient admissions only. We, however, feel confident this evaluation includes the vast majority of BSI episodes as the management of bacteremia at our institution is exclusively performed on the inpatient unit. Additionally, blood cultures obtained in our emergency department would be captured if the visit resulted in admission. However, BSI from blood cultures obtained at outside institutions with no subsequent positive cultures upon transfer and admission to our center would not be included in this analysis, as we could not ensure a complete data capture from this clinical scenario. Although our inability to account for these events may have contributed to an inflation of our mortality estimates from BSI, we estimate these events to be rare. Finally, while we demonstrated a clear association with mortality, we recognize that death in some of these patients may have been unrelated to BSI even when BSI is present.

## 5 Conclusion

In conclusion, in one of the largest reports to date, we have demonstrated that BSI remains a significant source of mortality in pediatric oncology patients. Further investigations into specific groups at high risk may further define BSI related mortality. Future studies are needed to reduce the incidence of BSI in these patients which would subsequently reduce mortality in this population and improve their overall long-term survival.

## Data availability statement

The raw data supporting the conclusions of this article will be made available by the authors, without undue reservation.

## Ethics statement

The studies involving human participants were reviewed and approved by Washington University Institutional Review Board. Written informed consent from the participants’ legal guardian/next of kin was not required to participate in this study in accordance with the national legislation and the institutional requirements.

## Author contributions

DW conceptualized and designed the study, collected and supervised the collection of data, drafted the initial manuscript and revised and reviewed the manuscript draft. MM collected data and revised and reviewed the manuscript draft. PR provided data validation and critically reviewed the manuscript for important intellectual content. RH aided in conceptualization of the study, provided supervision for the study, reviewed and revised the manuscript draft and provided important intellectual content. All authors approved the final manuscript as submitted and agree to be accountable for all aspects of the work. All authors contributed to the article and approved the submitted version.
